# Epitope Characterization and Variable Region Sequence of F1-40, a High-Affinity Monoclonal Antibody to Botulinum Neurotoxin Type A (Hall Strain)

**DOI:** 10.1371/journal.pone.0004924

**Published:** 2009-03-17

**Authors:** Miles C. Scotcher, Jeffery A. McGarvey, Eric A. Johnson, Larry H. Stanker

**Affiliations:** 1 United States Department of Agriculture, Agricultural Research Service, Western Regional Research Center, Albany, California, United States of America; 2 Department of Bacteriology, Food Research Institute, University of Wisconsin-Madison, Madison, Wisconsin, United States of America; The University of Queensland, Australia

## Abstract

**Background:**

Botulism, an often fatal neuroparalytic disease, is caused by botulinum neurotoxins (BoNT) which consist of a family of seven serotypes (A-H) produced by the anaerobic bacterium *Clostridium botulinum*. BoNT, considered the most potent biological toxin known, is a 150 kDa protein consisting of a 100 kDa heavy-chain (Hc) and a 50 kDa light-chain (Lc). F1-40 is a mouse-derived, IgG1 monoclonal antibody that binds the light chain of BoNT serotype A (BoNT/A) and is used in a sensitive immunoassay for toxin detection. We report the fine epitope mapping of F1-40 and the deduced amino acid sequence of the variable regions of the heavy and light chains of the antibody.

**Methods and Findings:**

To characterize the binding epitope of F1-40, three complementary experimental approaches were selected. Firstly, recombinant peptide fragments of BoNT/A light-chain were used in Western blots to identify the epitope domains. Secondly, a peptide phage-display library was used to identify the specific amino acid sequences. Thirdly, the three-dimensional structure of BoNT/A was examined *in silico*, and the amino acid sequences determined from the phage-display studies were mapped onto the three-dimensional structure in order to visualize the epitope. F1-40 was found to bind a peptide fragment of BoNT/A, designated L1-3, which spans from T125 to L200. The motif QPDRS was identified by phage-display, and was mapped to a region within L1-3. When the three amino acids Q138, P139 and D140 were all mutated to glycine, binding of F1-40 to the recombinant BoNT/A light chain peptide was abolished. Q-138, P-139 and D-140 form a loop on the external surface of BoNT/A, exposed to solvent and accessible to F1-40 binding.

**Conclusions:**

The epitope of F1-40 was localized to a single exposed loop (ß4, ß5) on the Lc of BoNT. Furthermore amino acids Q138, P139 and D140 forming the tip of the loop appear critical for binding.

## Introduction

Botulinum neurotoxins (BoNTs), the causative agents of botulism, are the most potent naturally-occurring toxins known [1]. Seven different serotypes of BoNT, designated A to G, are produced predominantly by strains of *Clostridium botulinum*, but also by some strains of *C. butyricum* and *C. baratii* [2]. Each serotype is further divided into one or more subtypes based upon strain of origin, resulting in over 40 immunologically distinct BoNT types [3]. BoNT/A (subtype A1), derived from the Hall strain of *C. botulinum* (ATCC 3502), is possibly the most widely studied and best understood of the BoNTs, and is used in this study.

BoNT is released following bacterial lysis as a 900 kDa complex in association with several non-toxic accessory proteins (NAPs) [4]. The BoNT/A holotoxin is comprised of a 100 kDa heavy chain (Hc) and a 50 kDa light chain (Lc), linked by a single disulphide bond. The Hc functions by binding nerve cells and facilitates the internalization of the Lc, a zinc endopeptidase that cleaves SNARE (soluble N-ethylmaleimide sensitive factor attachment receptor) proteins. This action prevents the release of acetylcholine from the neuron into the neuromuscular junction, ultimately resulting in flaccid paralysis of the muscle [5], [6]. The three-dimensional structure of the BoNT/A holotoxin has been determined at 3.3 Å resolution [7].

In mice, an LD_50_ of 10 pg per organism was reported for BoNT/A when administered by inter-peritoneal injection [8]. Early studies estimated a minimum human lethal dose (LD_100_) of BoNT/A at 1 ng per kg body mass when administered via inter-peritoneal injection [9], but it has since been recognized that the route of toxin exposure is critical in determining its lethality. For example, the human LD_100_ for BoNT/A is estimated at 1 µg/kg when administered orally, whereas via intravenous injection, the LD_100_ is 700-fold lower [10], [11].

Although outbreaks of foodborne BoNT poisoning are rare, their impact can be significant. In July 2007, four cases of foodborne botulism were linked to the consumption of hotdog chili sauce resulting in a recall of over 721,000 pounds of canned meat products in 49 states, at a projected cost to the manufacturer of $35 million [12]–[14]. BoNT has been used in, or developed for biological weapons since the 1930's, and is still considered a credible threat to national security today, being classified as a Class A bioterrorism agent by the CDC [15], [16]. It has been suggested that the most likely terrorist strategy would be to contaminate large batches of food or beverages with BoNT. One study described a mathematical model of where milk was intentionally adulterated [17].

These concerns demonstrate the need for tests to detect the presence of toxin in food. Such tests must be easily completed with a minimum amount of sample preparation, they should be sensitive, specific, and give accurate results in a timely manner. Despite the range of tests that have or are being developed, most require a skilled technician to prepare the sample and perform the test, and can take from several hours to four days to obtain results (for full reviews see [18], [19]). We recently reported the development and partial characterization of four high-affinity monoclonal antibodies (mAbs), designated F1-2, F1-5, F1-40 and F2-43, to BoNT/A from the Hall strain of *C. botulinum* [20]. Two of these mAbs, F1-2 and F1-40, were used to develop a sensitive sandwich-ELISA with a detection limit of 2 pg/mL, approximately 10-fold more sensitive than the mouse bioassay. Since F1-40 is used in this sensitive ELISA, molecular characterization and a detailed understanding of its binding properties are highly desirable to develop improved assays and enhance the predictive value of this diagnostic test. For example, F1-40 exhibits high affinity binding to the light chain of BoNT/A1, but other serotypes are not detected [20]. Understanding the molecular basis of this specificity would improve our ability to incorporate F1-40 into multianalyte assays able to detect more than a single serotype. In addition to development of improved diagnostics, mapping antibody epitopes onto the toxin is extending our understanding of the mechanisms by which antibodies, individually or in combinations, neutralize toxin [21], [22]. Preliminary data indicates that F1-40, by itself or as a component of a multi-antibody mixture is effective in protecting mice from BoNT/A [23]. Thus, characterization of the F1-40 epitope may help elucidate the mechanism by which it neutralizes the toxin. Furthermore, since F1-40, in a mouse model, was able to rescue animals after intoxication, cloning and sequencing the antibody heavy and light chain variable regions represents the first step in further engineering of this antibody.

In this article, we report the identification of the F1-40 epitope on the light chain of BoNT/A using three complementary experimental approaches. Firstly, antibody binding to recombinant peptide fragments of BoNT/A light-chain was investigated. Secondly, a peptide phage-display library was used to identify amino acid sequences that were bound by F1-40. Thirdly the three-dimensional structure of BoNT/A was examined *in silico*, and the ligands determined from the phage-display studies were mapped onto the three-dimensional structure in order to visualize a putative epitope. Mutagenesis studies were used to confirm the identified epitope. In an effort to further characterize mAb F1-40, the variable regions gene sequences of the heavy and light chains were determined, and the amino acid sequences of the complementary determining regions deduced.

## Methods

### Plasmid construction

Commercial enzymes (Phusion High-Fidelity DNA Polymerase, BamHI, XhoI, T4 polynucleotide kinase [3′ phosphatase minus], T4 DNA ligase [New England BioLabs Inc., Bethesda, MD]) were used according to the manufacturer's recommendation. Primers used were purchased from Integrated DNA Technologies (Coralville, IA) and are shown in Table 1. Plasmid construction and manipulation was performed in *Escherichia coli* TOP10 cells (Invitrogen, Carlsbad, CA) grown aerobically in Luria-Bertani (LB) medium at 37°C supplemented with 100 µg/mL ampicillin [24]. Plasmids or DNA were purified using the QuickClean 5M range of kits (GenScript Corp., Piscataway, NJ). All automated DNA sequencing was performed using the Big Dye Terminator Version 3.1 and XTerminator reagents, and a 3730 DNA Analyzer (Applied Biosystems, Foster City, CA).

**Table 1:** Primers.

Primer	Sequence	Constructs
LcF	GGATCCATGCCATTTGTTAATAAACAATTTAATTATAAAG	Lc, L1
LcR	CTCGAG **TTA**TTTAGAAGTTATTATCCCTCTTACAC	Lc, L2
L1R	CTCGAG **TTA**AAGTGACTCCTCAAAACCAAATG	L1
L2F	GGATCCGAAGTTGATACAAATCCTCTTTTAG	L2
GS-L	GGATCCGATATCAGCCATGGCC	L1-3, L1-4
GS-R	**TAA** CTCGAGCACCACCACCAC	L1-1,L1-2
L1b	TTCTGGTGGTGGATTTAAATCTCCTTC	L1-1
L1c	GCAAAACAAGTTCCAGTTTCATATTATGATTC	L1-4
L1d	ATCTATTGTACTTCCACCCCAAAATGG	L1-2
L1e	ACAGAATTAAAAGTTATTGATACTAATTGTATTAATGTG	L1-3
Lc-ΔL	ATTAGTATCAATAACTTTTAATTCTGT	Lc-Δ
Lc-ΔR	CTTAATCTAGTAATAATAGGACCCT	Lc-Δ
Lc-QPDL	ACCACCTATCACATTAATACAATTAGTATCAAT	Lc-QPD
Lc-QPDR	GGTGGTAGTTATAGATCAGAA	Lc-QPD
Lc-RSL	ACCATAACTACCATCTGGTTGTATCA	Lc-RS
Lc-RSR	GGAGAAGAACTTAATCTAGTAATAATA	Lc-RS
L-chainR	TCTAGAACTGGATGGTGGGAGATGGA	F1-40 L-chain cloning
H-chainR	TCTAGAACCTCCACACACAGGAACCAGTGGATAGAC	F1-40 H-chain cloning
L-chainF3	GATATCCACCATGGAGTCACAGACTCAGGTCTTTGTA	F1-40 L-chain cloning
H-chainF3	GATATCCACCATGGCTGTCTTGGGGCTGCTCTTCT	F1-40 H-chain cloning
M13F	GTAAAACGACGGCCAG	seq. pCR4 plasmids
M13R	CAGGAAACAGCTATGAC	seq. pCR4 plasmids
pGS-F	CAAATTGATAAGTACTTGAAATCC	seq. pGS-21a plasmids
pGS-R	GCTAGTTATTGCTCAGAGG	seq. pGS-21a plasmids

Sites for restriction enzymes BamHI (GGATCC), XhoI (CTCGAG), XbaI (TCTAGA) and EcoRV (GATATC) are shown underlined. Stop codons are shown in bold, either in the 5′ to 3′ (TAA) or 3′ to 5′ (TTA) orientation. The third column indicates which peptide fragments each primer was used to construct, or where the primer was used to clone the heavy and light chain variable regions of F1-40. Primers used only for sequencing are indicated by the abbreviation “seq.”

**Figure 1:**
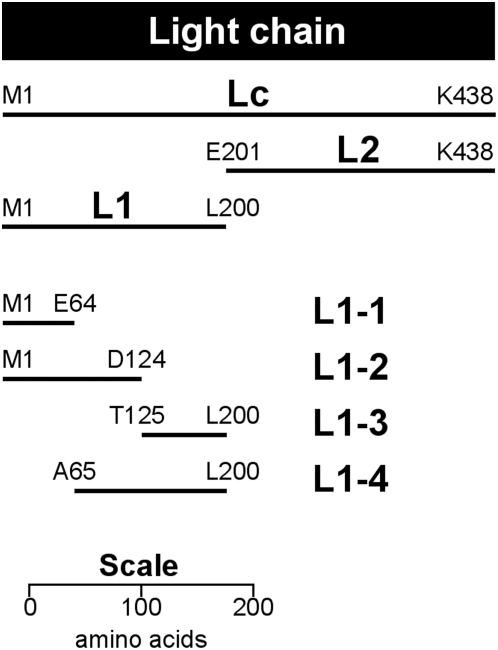
Peptide fragments of BoNT/A light chain. Diagram is drawn to scale to facilitate size and location comparison between peptide fragments. Peptide fragments were expressed as fusions to GST at the N-terminal. N- and C-terminal amino acids of each peptide fragment are indicated.

Total genomic DNA from *Clostridium botulinum* (Strain ATCC3502) was used as a template to amplify the fragments of the light chain (Lc, L1, L2) using the primers indicated (see Figure 1 and Table 1). Stop codons (TAA) were introduced when not present within the genomic DNA of the cloned region. All subsequent BoNT/A DNA fragments were cloned into plasmid pCR4-TOPO (Invitrogen) to allow sequencing using primers M13F and M13R. The pCR4-derived plasmids were then digested using BamHI and XhoI, the BoNT/A fragment was purified and ligated into BamHI- and XhoI-digested pGS-21a (Genscript) to yield the correspondently named pGS plasmid (e.g. pGS-L1 for fragment L1). All pGS-21a-derived plasmids were sequenced using primer pGS-F and pGS-R, to confirm the correct integration of the BoNT/A fragment into the vector. The BamHI and XhoI cloning sites of pGS-21a are located downstream of glutathione-S-transferase (GST), under the control of the T7 promoter.

Plasmids pGS-L1-1 through pGS-L1-4 were constructed by PCR using plasmid pGS-L1 as a template. Primers were used to amplify outwards from the L1 region, thus eliminating internal pieces of L1 (see Figure 1). The PCR product was gel purified, treated with T4 polynucleotide kinase then self-ligated to form an intact plasmid. The mutant plasmids pGS-Lc-Δ, pGS-Lc-QPD and pGS-Lc-RS (see Table 2) were constructed by PCR in an identical manner, using plasmid pGS-Lc as a template, and the primers shown in Table 1.

**Table 2:** Mutations made in the putative F1-40 epitope region on plasmid pGS-Lc.

Vector	Light chain, from T-125 to Q-162
pGS-Lc	TELKVIDTNCINVI**QPD**GSY**RS**EELNLVIIGPSADIIQ
pGS-Lc-Δ	TELKVIDTN---------------LNLVIIGPSADIIQ
pGS-Lc-QPD	TELKVIDTNCINVI**GGG**GSYRSEELNLVIIGPSADIIQ
pGS-Lc-RS	TELKVIDTNCINVIQPDGSY**GG**EELNLVIIGPSADIIQ

All four vectors shown above harbored and expressed the entire BoNT/A light chain (P-1 to K-438), but only the mutated region (T-125 to Q-162) is shown here. All other amino acids within the light chain were not mutated. Dashes indicate that amino acids have been deleted. Single amino acid mutations are indicated in bold.

### Expression and purification of GST-fusion proteins

All pGS-21a-derived plasmids were transformed into *E. coli* BL21-CodonPlus (DE3)-RIPL cells (Stratagene, La Jolla, CA), and grown aerobically on LB agar at 37°C supplemented with 100 µg/mL ampicillin and 75 µg/mL streptomycin. Single colonies were grown overnight in LB containing the same antibiotics and 0.5% glucose, to minimize uninduced expression of the fusion protein [25]. An inoculum of 6 mL was added to 600 mL 2YT medium (1% tryptone, 1.6% yeast extract, 0.5% NaCl) containing the same antibiotics and glucose, and the culture was grown aerobically at 30°C with 200 rpm agitation to an OD_600_ of ∼0.6. Expression was induced with 1 mM IPTG (isopropyl ß-D-1 thiogalactopyranoside), the culture was grown overnight at 18°C and cells were harvested by centrifugation at 10000×*g* for 10 min.

The cell pellet was suspended in 10 mL lysis solution (PBS [10 mM phosphate buffer, 138 mM NaCl, 2.7 mM KCl, pH 7.4], 1 mM PMSF (phenylmethanesulphonylfluoride), 0.2 mg/mL lysozyme, 1× CelLytic-B Cell Lysis Reagent, 0.5 µL Benzonase Nuclease [Sigma-Aldrich Inc., St. Louis, MO]) and incubated at 37°C for 10 min with 200 rpm agitation, then placed immediately on ice. The lysate was clarified by centrifugation at 12800×*g* for 15 min, then loaded directly onto a column of High-Affinity GST Resin (1 mL bed volume; Genscript) that had been equilibrate with 20 bed volumes ice-cold PBS containing 1 mM PMSF. The column was washed with 30 bed volumes ice-cold PBS-PMSF and then eluted with 15 bed volumes of elution buffer (10 mM reduced glutathione, 50 mM Tris, pH 8.0) in fractions of 1 mL.

### Electrophoresis and Western blots

All gel electrophoresis equipment, buffers, gels and nitrocellulose membranes were purchased from PAGEgel (San Diego, CA). Samples of 40 µL (25 µL protein sample, 10 µL 4× gel buffer, 4 µL 1 M dithiothreitol) were heated for 10 min at 70°C, then loaded onto a 10% gel and separated by electrophoresis at 175 V (constant) for 80 min. For protein visualization, gels were silver stained using the Silver Express kit (Invitrogen) according to manufacturer's instructions. For Western blotting, the gel was subjected to horizontal blotting onto a nitrocellulose membrane using a PAGEgel transfer cell (180 mA constant, 100 min). Membranes were incubated in blocking buffer (1× PBS-T [PBS plus 0.05% Tween-20], 3% non-fat dried milk) for 1 hr at room temperature with gentle agitation. Membranes were incubated for 1 hr at 37°C with gentle agitation in primary antibody (F1-40 or a rabbit polyclonal to *Clostridium botulinum* A toxoid # 20641 [Abcam Inc., Cambridge, MA]) diluted 5 µg/mL in 10 mL of blocking buffer. Membranes were washed 4 times in PBS-T. Membranes were incubated for 1 hr at room temperature with gentle agitation in secondary antibody diluted 1∶5000 in blocking buffer. Peroxidase-conjugated, goat anti-mouse IgG #A4416 was used to detect F1-40; peroxidase-conjugated, goat anti-rabbit IgG #A6154 was used to detect the polyclonal antibody (Sigma-Aldrich). Membranes were washed 4 times in PBS-T, then SuperSignal West Dura Extended Duration Substrate (Pierce, Rockford, IL) was added according to manufacturer's instructions. Membranes were visualized using a Fluorchem SP unit (Alpha Innotech Corp., San Leandro, CA).

### Competition ELISA

Black 96-well plates were coated with the Lc-GST fusion peptide by adding 100 µL per well of a 4 µg/mL solution of Lc peptide in 0.05 M carbonate buffer (pH 9.6) and incubating overnight at 4°C. Plates were then blocked by adding 300 µL blocking buffer (1× PBS-T [PBS plus 0.05% Tween-20], 3% non-fat dried milk) to each well and incubating for 1 hr at room temperature. Wells were filled with 100 µL blocking buffer containing decreasing concentrations (120, 60, 30, 15, 7.5, 3.75, 1.88, 0.94, and 0 µg/mL) of a fusion peptide in solution (Lc, Lc-Δ, Lc-QPD or Lc-RS) and then 100 µL of a 5 µg/mL solution of F1-40 was immediately added to each well. Plates were incubated overnight at 4°C with gentle agitation, then washed 12 times in PBS-T. Next, 200 µL of peroxidase-conjugated goat anti-mouse IgG #A4416 (Sigma-Aldrich) diluted 1∶5000 in blocking buffer was added to each well and the plate incubated for 1 hr at room temperature with gentle agitation. Plates were washed 12 times in PBS-T. Wells were filled with 100 µL of SuperSignal ELISA Femto Maximum Sensitivity Substrate (Pierce) and incubated for 3 min at room temperature with gentle agitation. Luminescent counts were recorded using a Wallac Victor 2 Multilabel Counter (PerkinElmer Inc., Waltham, MA). The percentage inhibition of binding was calculated by the formula (1-B/B_0_)×100, where B = luminescent counts at each concentration of fusion peptide in solution and B_0_ = luminescent counts at 0 µg/mL fusion peptide in solution.

### Phage Display

The Ph.D.-C7C Phage Display Peptide Library Kit (New England BioLabs) was used to pan against 60 mm polystyrene dishes coated with antibody F1-40, to identify peptide ligands to F1-40, according to manufacturer's instructions. The M13 phage displays a randomized amino acid heptamer between two cysteine residues on the pIII minor coat protein. The cysteine residues form a disulphide bond, resulting in the heptamer being presented as constrained loop. Four series of panning and three phage amplification stages were carried out, prior to the growth and sequencing of individual plaques of the phage. A total of twelve clonal plaques from the 4^th^ pan were picked for sequencing.

### Cloning and sequencing of antibody variable regions

The method used here for the cloning and sequencing of variable regions for antibody heavy and light chains is based upon the method described fully in Current Protocols in Immunology [26]. Significant changes from this methodology are described here.

Hybridoma cells for F1-40 production were grown as previously described [20]. Culture volumes of 5 mL were centrifuged at 12000×g for 1 min to collect the cells. mRNA was purified from the cells using Trizol Reagent (Invitrogen), according to manufacturer's instructions. cDNA was transcribed into mRNA using primers H-chainR and L-chainR, and AMV reverse transcriptase according to manufacturer's instructions (Promega, Madison, WI) The cDNA was amplified by PCR using Phusion DNA polymerase and the appropriate pairs of primers (H-chain R and F3; L-chain R and F3). The cDNA was gel purified, then treated using the MasterTaq Kit (Eppendorf North America, Westbury, NY) according to manufacturer's instructions to add A-overhangs to the DNA. The cDNA was then cloned into plasmid pCR4-TOPO, and transformed into TOP10 cells. Six individual colonies of *E. coli* harboring pCR4-H-chain and pCR4-L-chain were picked, grown and prepared for sequencing as described earlier.

### Computer tools

All DNA and amino acid sequence analysis was carried out using the tools of the Biology Workbench, Version 3.2 (http://workbench.sdsc.edu San Diego Supercomputer Center, University of California, San Diego, CA). The three-dimensional diagrams of BoNT/A were generated using the crystal structure of BoNT/A [7]) manipulated with the MBT Protein Workshop (RCSB Protein Data Bank, www.rcsb.org/pdb).

## Results

### Binding of F1-40 to peptide fragments of the BoNT/A light chain

**Figure 2:**
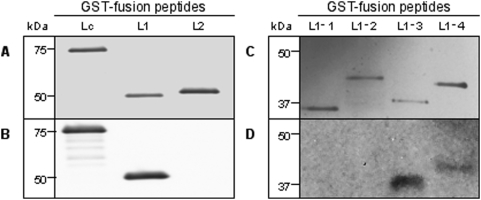
Binding of F1-40 to peptide fragments of BoNT/A light chain. A and C, Light chain fragment-GST fusion peptides separated by gel electrophoresis and stained with Silver Stain. B and D, Corresponding Western Blots using F1-40 for primary detection. Fusion peptide size is indicated in kDa.

Seven recombinant peptide fragments of BoNT/A light chain (Lc, L1, L2, L1-1, L1-2, L1-3, L1-4) are shown in Figure 1. These recombinant peptides were expressed as fusions to glutathione-S-transferase (GST). The relative molecular weights (rMW) of these recombinant peptide-GST proteins, observed by SDS-PAGE and silver staining, are shown in Figure 2, panels A and C. In each case, the rMW corresponds to that predicted for the recombinant peptide-GST protein. Results from Western blot analyses are shown in panels B and D. Clearly, F1-40 bound to the light-chain fragment (Lc) and to subfragment L1, but binding to L2 was not detected (Figure 2, Panel B). The L1 peptide fragment corresponds to amino acids M1 to L200. This region was further subdivided by generating four additional recombinant peptide fragments L1-1 through L1-4 (Figure 1). In Western blotting experiments, F1-40 was observed to bind two of these recombinant peptide fragments, L1-3 and L1-4 (Figure 2, Panel D). In contrast, binding to L1-1 and L1-2 was not detected. These results, particularly antibody binding to L1-3, suggest that the epitope for F1-40 resides between amino acids T125 and L200.

### Fine epitope maping of F1-40 using heptamer peptide ligands identified by phage display

**Figure 3:**
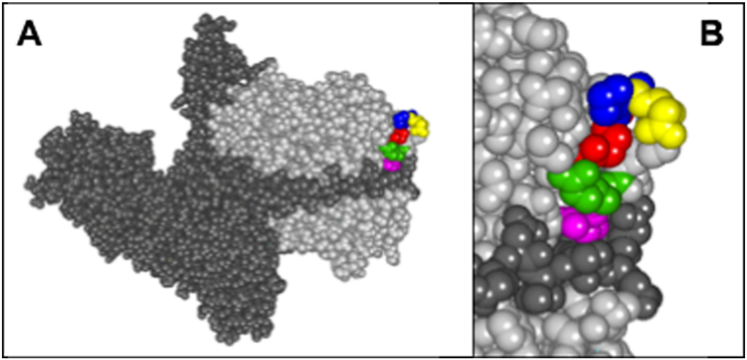
Location of the QPDRS motif within the BoNT/A holotoxin. A. Space-filled diagram of the three-dimensional model of the complete BoNT/A holotoxin showing the heavy (dark gray) and light (light gray) chains. QPDRS motif is the colored region within the light chain. B. Enlarged view of Q139 (red), P140 (blue), D141 (yellow), R145 (green) and S146 (pink) [7].

In an effort to further localize the epitope for F1-40, binding to a random phage display library of heptamer peptides was evaluated. Following four rounds of panning, twelve clonal plaques picked for sequencing. One plaque failed to yield sequence, and the remaining eleven sequences of the heptamer peptide ligands for F1-40 are shown, with their frequency of occurrence, in Table 3. The most common motif, SSAFYPK, found in eight of the eleven plaques sequenced, did not readily map to a putative epitope region on the L1-3 peptide fragment, or to any other region on the light chain of BoNT/A and most probably represents a mimotope. In contrast, the motif QPDRS, common to the remaining three heptamer sequences, when mapped onto the three-dimensional structure of BoNT/A [7] corresponded to the turn (Q139, P140, D141) between ß4–ß5 and the first two amino acids (R144, S145) of ß5. While R144 and S145 are separated from amino acids QPD (the very tip of the loop) they are brought into close proximity to each other by the tertiary structure of the toxin (See Figure 3).

**Table 3:** Amino acid sequences identified using phage display against F1-40.

Heptamer sequence	Frequency
S S A F Y P K	8
T R Q P D R S	1
T L Q P D R S	1
S L Q P D R S	1

The sequences of the heptamer peptide ligands to mAb F1-40 were identified using the Ph.D.-C7C Phage Display Peptide Library Kit (New England BioLabs Inc.) to pan against mAb F1-40. Twelve clonal phage plaques were sequenced, one failed to yield sequence, and the frequency of the heptamer sequences observed is shown.

### Binding of F1-40 to mutants of the Lc peptide fragment

**Figure 4:**
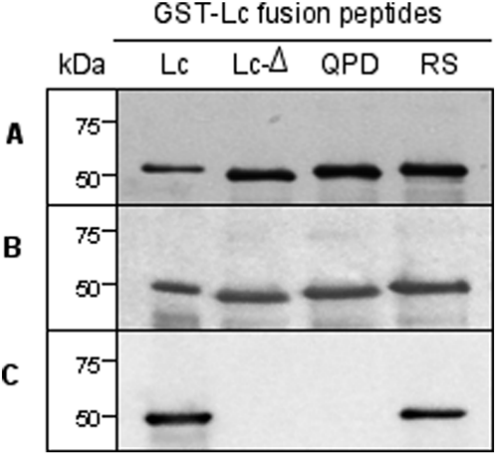
Binding of F1-40 to the Lc peptide fragment mutants. A., Lc peptide fragment mutants separated by gel electrophoresis and stained with Silver Stain. B, Western Blot using rabbit polyclonal to *Clostridium botulinum* A toxoid # 20641 (Abcam Inc.) for primary detection. C, Western Blot using F1-40 for primary detection. Peptide size is indicated in kDa.

We developed mutant recombinant Lc peptides carrying specific amino acid deletions or substitutions within the region identified via phage display (Table 2). The region spanning from C134 to E148 (ß4–ß5) forms a looped structure, the tip of which corresponds to Q139-P140-D141. The entire loop was deleted from the recombinant peptide Lc to form mutant peptide Lc-Δ. In mutant peptide Lc-QPD the amino acids Q139, P140 and D141 were all mutated to glycine. In mutant peptide Lc-RS the amino acids R145 and S146 were both mutated to glycine. Panel A in Figure 4 shows a silver stained gel of the Lc peptide fragment and the three mutant peptides. As a control, a polyclonal anti-BoNT/A toxoid antiserum was used in a Western blot, which clearly bound the Lc peptide and all three mutant peptides (Panel B). Binding of F1-40 to these mutant peptides is shown by Western blot in Panel C. F1-40 bound the wild-type Lc peptide and the mutated peptide Lc-RS, but binding to mutated peptides Lc-Δ and to Lc-QPD was not detected. These data demonstrate that the mutation of the Q139, P140, D141 motif to GGG decreases F1-40 binding to a level which was not detected by Western blotting.

### Competition ELISA

**Figure 5:**
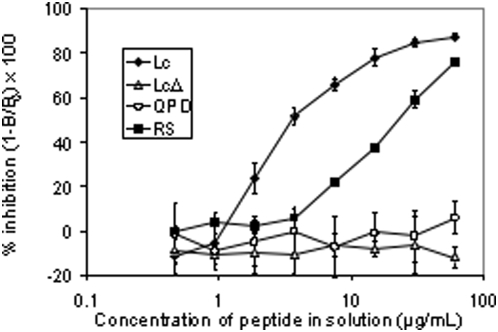
Competition ELISA for F1-40 by Lc peptide fragment mutants in solution. The percent inhibition of binding of mAb F1-40 to immobilized Lc peptide fragment on a 96-well plate by various peptide fragments in solution (♦, Lc; ▵ Lc-Δ; ○ Lc-QPD; ▪ Lc-RS) was calculated by the formula (1-B/B_0_)×100, where B = luminescent counts at each concentration of fusion peptide in solution, and B_0_ = luminescent counts at 0 µg/mL fusion peptide in solution. Data is presented±1 standard error, n = 4.

Competition ELISA assays were performed to better quantify the differences of antibody binding to the mutant peptides observed in the above Western blotting experiments. Results from these experiments are shown in Figure 5. Peptide Lc was the most effective at competing binding of F1-40 to immobilized Lc, with 50% inhibition achieved at a concentration of ∼3.5 µg/mL. Mutant peptide Lc-RS was the next most effective competitor resulting in 50% inhibition of control activity at ∼24 µg/mL, roughly 7-fold higher than that observed for the recombinant Lc peptide. Finally, mutants Lc-Δ and Lc-QPD did not compete for F1-40 binding under these conditions, even at the highest concentration of peptide used (60 µg/mL).

### F1-40 heavy and light chain variable region sequences

**Figure 6:**
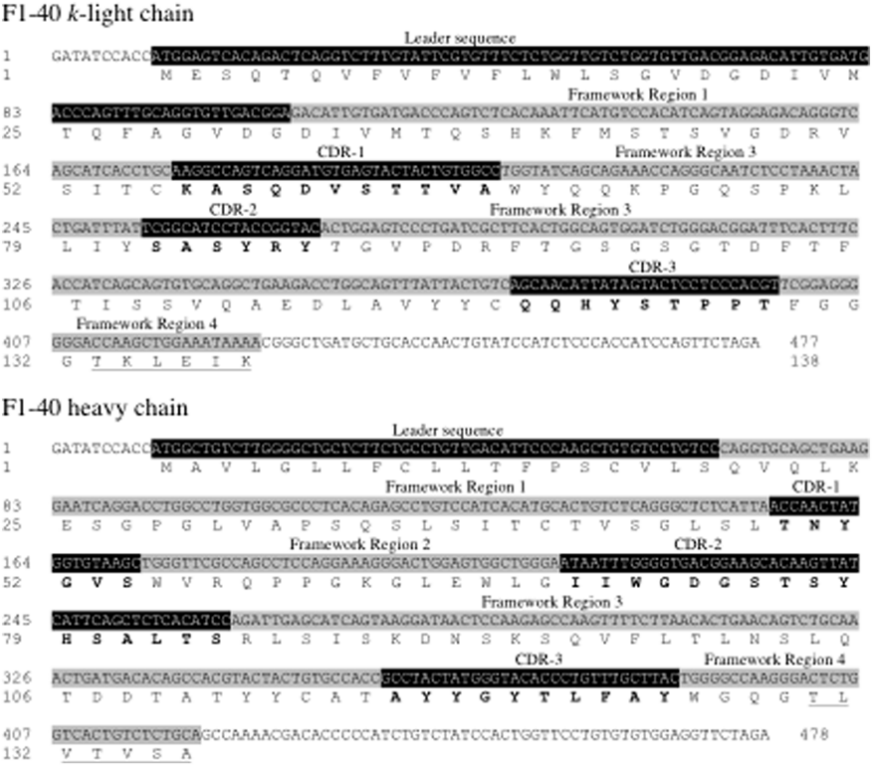
Nucleotide and amino acid sequences for the variable regions of F1-40 *k*-light and heavy chains. The nucleotide sequences for the entire cloned regions are shown, with the deduced amino acids from the leader sequence to the end of the fourth framework region. The leader sequence and three complementarity determining regions (CDRs) are shown highlighted in black, with the amino acids of the CDRs shown in bold. The four framework regions are shown highlighted in light gray, with the amino acids of the J-regions of both light and heavy chains shown underlined, within the fourth framework region. EMBL online Accession #s FM177889 & FM177890.

The cDNA sequences of the heavy and light chain variable regions for F1-40 are shown in Figure 6. The nucleotide sequence of the entire cloned regions is shown, with the corresponding amino acids shown from the start of the leader peptide to the end of the fourth framework region of each chain. The leader sequences, framework regions, complementarity determining regions (CDRs) and J-regions were identified by inspecting the alignment of the F1-40 heavy and light chains to other antibody sequences [26]–[30]. The nucleotide sequence reported was identical across the six individual colonies of *E. coli* harboring either pCR4-L-chain or pCR4-H-chain that were analyzed. EMBL Nucleotide Sequence Database accession numbers for these sequences are FM177889 and FM177890, for the light and heavy chains, respectively.

## Discussion

The epitope of mAb F1-40 was mapped to the exposed loop located between ß4-ß5 on the light chain of BoNT/A (Figure 3). The amino acids QPDRS, identified by phage display experiments mapped to this region, Q139, P140, and D141 form the tip of the loop and R145 and S146 are brought into close proximity by the tertiary structure of BoNT (Figure 3).

Previous studies in our laboratory [20] demonstrated that mAb F1-40 bound the light chain domain (Lc) of BoNT. To more clearly define the binding epitope, the Lc domain of BoNT/A as well as smaller fragments were cloned into *E. coli* and expressed as GST fusion proteins (Figure 2). Western blotting experiments measuring antibody binding to these recombinant peptide fragments of toxin Lc demonstrated that the epitope for F1-40 was contained within a 76 amino acids region from T125 to L200. Fine epitope mapping by analysis of binding sequences identified in phage display experiments suggested that a peptide ligand containing the amino acid motif QPDRS might constitute part or all of the F1-40 epitope. When these amino acids were mapped onto the three-dimensional crystal structure of BoNT/A [7], they formed a compact, almost continuous sequence corresponding to an external loop (ß4, ß5) region of the molecule (Figure 3). Amino acids Q139, P140, and D141 form the tip of the loop and are separated from residues R145 and S146 by three intervening amino acids. However, the BoNT/A tertiary structure brings these amino acids R145 and S146 into close proximity of Q139, P140, and D141. Replacement of R145 and S146 with glycine residues had no obvious effect on F1-40 binding as illustrated by Western blot, but competition ELISA experiments revealed a 7-fold decrease in the relative affinity of mutant Lc-RS for F1-40 compared to the recombinant Lc peptide. These data suggest that under the conditions of SDS-PAGE, binding is not visibly altered by mutating R145 and S146. However, the competiton ELISA data demonstrated that R145 and S146 do contribute in part to the binding of F1-40 to the light chain of BoNT/A, under the solution conditions of competition ELISA. Mutation of Q139, P140 and D141 to glycine residues or eliminating the entire loop (mutant LcΔ) abolished binding of F1-40 to the Lc peptide in both the competition ELISA and Western blotting experiments, indicating that the QPD triad is necessary for the binding of F1-40 to the light chain of BoNT/A.

We have previously shown that binding of F1-40 to BoNT serotypes B through G is undetectable by ELISA [20]. All three amino acids, Q139, P140, D141, forming the turn of the exposed loop (ß4–ß5) are exposed to solvent (Figure 3). Analysis of the amino acid sequence and three-dimensional structure of the light chain of BoNTs B through G reveals that although a loop is formed in a similar position on the light chain in all other BoNT types (except BoNT/C – there is no crystal structure available), none possess the QPD motif in its entirety [31]–[35]. It is highly probable that the inability of F1-40 to bind the other serotypes of BoNT is due to the absence of a complete QPD motif. Interestingly, BoNT/G possesses Q144 and P145 (corresponding to Q139 and P140) but a glycine not aspartate residue at position 146 (corresponding to D141), yet it remains unable to bind F1-40, leading to speculation about the possible role that D141 might play in determining the specificity of F1-40 to BoNT/A [20], [34].

Using a series of overlapping synthetic peptides Dolimbek et al. [36] successfully mapped continuous regions of BoNT/B recognized by antibodies derived from human, horse and mouse sera. Such experiments can identify immunodominant areas of a protein and they have often been used to identify antibody binding epitopes. However, this approach only detects linear epitopes and overlooks complex, conformational epitopes. Using phage display libraries expressing BoNT Hc gene fragments, the epitopes of two Hc-specific anti-BoNT antibodies were identified [37]. Levy et al. [26] reported the effective use of a randomly mutated BoNT/A heavy chain library, displayed on the surface of yeast cells, to identify single amino acids important in the epitopes of anti-BoNT/A monoclonal antibodies. In the absence of such libraries for the BoNT/A light chain, we have nonetheless been able to characterize the epitope of F1-40 to a similarly high resolution. We expect our experimental approach to increase the likelihood of identifying epitopes of other antibodies in the future, and to facilitate their characterization at the single amino acid level.

Although the phage display analysis yielded the sequence QPDRS, it also provided the anomalous motif SSAFYPK in eight of the eleven sequenced plaques. Unlike the sequence QPDRS, this second sequence could not be mapped onto the light chain of BoNT/A, and it probably represents a mimotope of the F1-40 epitope. Mimotopes are commonly identified by phage display, and this faculty has been widely exploited to generate potential peptide vaccine candidates [22], [38]–[39]. When using phage display to search for an epitope, multiple mimotopes that bind a monoclonal antibody can be selected, and it is necessary to examine every mimotope to identify an epitope region [40], [41]. Supporting evidence defining an epitope, such as the Lc peptide mutagenesis data presented in this study, must be included in the analysis before assigning an epitope to an antibody. The assignment of an epitope motif to F1-40 including Q139, P140, D141, R145 and S146 is consistent with the BoNT serotype A specificity of F1-40.

Sequence analysis of the cDNA derived from the mRNA coding for the heavy and light chains of F1-40 reveals features that are typical of mouse monoclonals antibodies [28], [30], [42]. On the *k*-light chain, the section GVDGDIVMTQ from G29 to Q38 is a repeat of a preceding section from G17 to Q26, and forms the junction between the leader sequence and first framework region of the mature light chain. This repeat is not always present in *k*-light chains. The J-region of the heavy chain, TLVTVSA, is type 3 and the J-region of the *k*-light chain, TKLEIK, is type 2 [27].

In conclusion, we have localized the epitope of anti-BoNT/A mAb F1-40 to the exposed loop between ß4-ß5 regions. Both the our phage display and mutant-binding data suggest that amino acids Q139, P140, and D141, located at the tip of this loop, are critical for antibody binding. In addition, we report the cDNA and deduced amino acid sequences the variable and J-regions of the antibody's heavy and light chains. The epitope assignment to a specific loop not only provides important insight into the nature of the interaction between F1-40 and BoNT/A, but also is highly useful for the future development of F1-40 as an integral component of a test for BoNT/A contamination of food.
