# Correction: Epitope Characterization and Variable Region Sequence of F1-40, a High-Affinity Monoclonal Antibody to Botulinum Neurotoxin Type A (Hall Strain)

**DOI:** 10.1371/annotation/8d83a0a4-4dd0-4c57-ad94-304130f565e6

**Published:** 2009-10-09

**Authors:** Miles C. Scotcher, Jeffery A. McGarvey, Eric A. Johnson, Larry H. Stanker

The second to the last paragraph of the Discussion Section is incorrect. This paragraph should read: Sequence analysis of the cDNA derived from the mRNA coding for the heavy and light chains of F1-40 reveals features that are typical of mouse monoclonals antibodies [28], [30], [42]. The J-region of the heavy chain, TLVTVSA, is type 3 and the J-region of the k-light chain, WTFGGGTKLEIK, is type 1 [27].

