# Correction: Epitope Characterization and Variable Region Sequence of F1-40, a High-Affinity Monoclonal Antibody to Botulinum Neurotoxin Type A (Hall Strain)

**DOI:** 10.1371/annotation/47f9bc3f-7c79-453c-85e3-588bc3056951

**Published:** 2009-10-15

**Authors:** Miles C. Scotcher, Jeffery A. McGarvey, Eric A. Johnson, Larry H. Stanker

A reference was omitted from the second paragraph under the subheading “Cloning and sequencing of antibody variable regions” in the Methods section. The text of the paragraph is also incorrect.

A correct version of the paragraph with the reference reads: To clone the kappa-light chain of F1-40, mRNA was converted to cDNA using an oligo dT 18-mer primer, and the light chain was amplified by PCR using primers 3’Kc and 5’Mk, as previously described (Wang et al., 2000). The PCR product was gel-purified and treated with polynucleotide kinase (New England BioLabs, Bethesda MD). Circularized vector pCR2.1 (Invitrogen, Carlsbad CA) was digested with EcoRV and treated with calf intestinal phosphatase (New England BioLabs). The PCR product was ligated into vector pCR2.1, purified and sequenced as described.

The reference is: Wang Z, Raifu M, Howard M, Smith L, Hansen D, et al. (2000) Universal PCR amplification of mouse immunoglobulin gene variable regions: the design of degenerate primers and an assessment of the effect of DNA polymerase 3’ to 5’ exonuclease activity. J Immunol Methods 233: 167-177.

