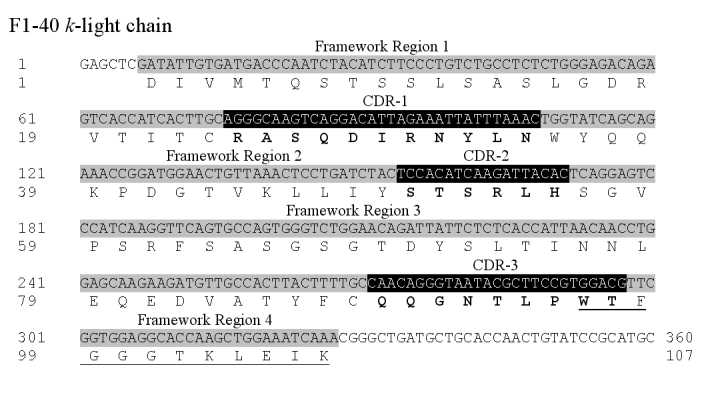# Correction: Epitope Characterization and Variable Region Sequence of F1-40, a High-Affinity Monoclonal Antibody to Botulinum Neurotoxin Type A (Hall Strain)

**DOI:** 10.1371/annotation/6359a6c2-90f8-4f7e-ad86-5bac5d62a7fe

**Published:** 2009-11-02

**Authors:** Miles C. Scotcher, Jeffery A. McGarvey, Eric A. Johnson, Larry H. Stanker

The kappa-light chain sequence, submitted as the first part of Figure 6, is incorrect. This sequence actually represents an extraneous light chain, produced by the Sp2/0 myeloma cell lines that were used to make the hybridomas that ultimately expressed F1-40. We have since identified a second kappa-light chain in the F1-40–expressing hybridomas which is the actual kappa-light chain for F1-40. No other kappa-light chain has been identified in the F1-40-producing hybridomas. Please view a corrected version here: 

**Figure:**